# SAC-MS: Joint Slice Resource Allocation, User Association and UAV Trajectory Optimization with No-Fly Zone Constraints

**DOI:** 10.3390/s25185833

**Published:** 2025-09-18

**Authors:** Geng Chen, Fang Sun, Gang Jing, Tianyu Pang

**Affiliations:** 1 College of Electronic and Information Engineering, Shandong University of Science and Technology, Qingdao 266590, China; gengchen@sdust.edu.cn (G.C.); fangsun0820@163.com (F.S.); ty_pang@163.com (T.P.); 2 College of Electrical Engineering and Automation, Shandong University of Science and Technology, Qingdao 266590, China

**Keywords:** SAGIN, user association, slice resource allocation, UAV trajectory, matching game, SAC, SCA

## Abstract

With the rapid growth of user service demands, space–air–ground integrated networks (SAGINs) face challenges such as limited resources, complex connectivity, diverse service requirements, and no-fly zone (NFZ) constraints. To address these issues, this paper proposes a joint optimization approach under NFZ constraints, maximizing system utility by simultaneously optimizing user association, unmanned aerial vehicle (UAV) trajectory, and slice resource allocation. Due to the problem’s non-convexity, it is decomposed into three subproblems: user association, UAV trajectory optimization, and slice resource allocation. To solve them efficiently, we design the iterative SAC-MS algorithm, which combines matching game theory for user association, sequential convex approximation (SCA) for UAV trajectory, and soft actor–critic (SAC) reinforcement learning for slice resource allocation. Simulation results show that SAC-MS outperforms TD3-MS, DDPG-MS, DQN-MS, and hard slicing, improving system utility by 10.53%, 13.17%, 31.25%, and 45.38%, respectively.

## 1. Introduction

With the advent of the sixth-generation (6G) wireless communication era, the paradigm of ubiquitous connectivity and support for diverse vertical industries has placed unprecedented demands on network capabilities. To meet these heterogeneous service demands, network slicing has emerged as a promising solution. It enables the partitioning of a shared physical network into multiple virtual networks based on different service types, with each network independently optimizing its resources and control strategies [1,2]. Driven by the rapid proliferation of high-bandwidth applications such as Internet of Things (IoT) devices and intelligent terminals, global mobile data traffic has increased dramatically. Traditional terrestrial networks are increasingly unable to meet the needs of wide-area coverage, high-speed access, and ultra-low latency, especially in remote or disaster-stricken regions. In these areas, terrestrial base stations are limited by geographical constraints and high deployment costs, resulting in significant coverage gaps. Furthermore, in urban hotspots or large-scale event scenarios, severe network congestion can significantly degrade the quality of service.

To address these challenges, the space–air–ground integrated network (SAGIN) has emerged as a promising architecture for next-generation wireless networks [3,4,5]. Specifically, low earth orbit (LEO) satellites can offer seamless connectivity in remote areas; however, the long communication distances between satellites and users hinder their ability to meet low-latency requirements [6]. In contrast, UAVs, acting as aerial base stations, offer flexible and rapid deployment and benefit from line-of-sight (LoS) communication, making them well-suited to complement terrestrial infrastructures [7,8]. By integrating satellites, unmanned aerial vehicles UAVs, and terrestrial networks, SAGIN enhances edge computing capabilities and improves network resilience, thereby alleviating the burden on traditional networks. This architecture is particularly well-suited for scenarios involving emergency response, coverage of remote areas, and dynamic service demands.

In practical deployment scenarios, UAVs, acting as mobile aerial base stations, provide communication and computing services to ground users. The design of UAV flight trajectories plays a critical role in determining the overall system performance. To achieve wide-area coverage and efficient service provisioning for ground users, it is essential to plan UAV flight paths carefully. A substantial body of research has been devoted to UAV trajectory optimization in multiple access systems [9,10,11], multi-UAV cooperative networks [12], and relay-based communication systems [13], yielding valuable insights.

However, in real-world environments, UAV trajectories are strictly constrained by regulatory and geographical factors. High-risk areas such as airports, power transmission lines, and military zones are often designated as no-fly zones (NFZs) [14], which must be explicitly considered in UAV trajectory planning. In recent years, several studies have begun incorporating NFZ constraints into trajectory optimization frameworks to enhance the safety and feasibility of UAV operations [15,16]. On the other hand, within the architecture of space–air–ground integrated networks, the way ground users select the most suitable access point—whether a terrestrial base station, UAV, or satellite—significantly impacts overall network performance. Moreover, given the limited communication and computing resources available at each access node, efficiently allocating these resources to meet heterogeneous service demands remains a key challenge requiring urgent attention.

Given the aforementioned challenges, this study develops a space–air–ground integrated network architecture with NFZ constraints. The proposed framework jointly optimizes user association, slice resource allocation, and UAV trajectory planning to meet the diverse service requirements of ground users. The main contributions of this work are summarized as follows:

- To meet the customized demands of diverse services under resource-constrained conditions, this paper introduces a dynamic radio access network slicing mechanism within the architecture of a SAGIN, subject to NFZ constraints. This design aims to enable on-demand allocation and efficient management of communication resources.
- We formulate a joint optimization problem that integrates user association, slice resource allocation, and UAV trajectory optimization, aiming to maximize the system utility, defined as the difference between the total system gains and the system costs. It is then decomposed into three subproblems: user association, UAV trajectory optimization and slice resource allocation.
- We propose the SAC-MS algorithm to solve the joint optimization problem. Specifically, a many-to-one matching game is adopted to achieve stable user–base station association, the SCA method is employed to transform the non-convex UAV trajectory optimization problem into convex subproblems, and a deep reinforcement learning-based algorithm is introduced for adaptive slice resource allocation in dynamic networks.
- Simulation results show that the proposed algorithm improves system utility by 10.53%, 13.17%, 31.25%, and 45.38% compared to the benchmark algorithms (TD3-MS, DDPG-MS, DQN-MS, and Hard_slicing), respectively.

The remainder of this paper is organized as follows. Section 2 provides a comprehensive review of related works on SAGIN, UAV trajectory optimization, user association, and slice resource allocation. Section 3 describes the system model, including the network model, UAV mobility model, NFZ model, communication model, and computation model. Section 4 formulates the joint optimization problem. Section 5 presents the proposed joint algorithm for user association, UAV trajectory optimization, and resource allocation. Section 6 analyzes the simulation results. Finally, Section 7 concludes the paper. To ensure clarity, we summarize all symbols and their definitions in Table 1 and provide the explanations of Acronyms in Table 2.

## 2. Related Works

To meet the growing demand for wireless services, the space–air–ground integrated network (SAGIN) has emerged as a pivotal architecture for enhancing connectivity and service quality by integrating satellites, UAVs, and terrestrial infrastructures. While extensive research has been conducted on its individual components, a significant gap remains in holistically integrating these elements under practical constraints [17]. 

Regarding SAGIN architectures, studies have made progress in supporting seamless coverage and massive connectivity. For instance, Mao et al. [18] introduced a space–aerial-assisted hybrid cloud–edge computing framework to minimize computation delay, while Cheng et al. [19] developed a SAGIN-based computing architecture for offloading. Others have focused on service function chaining [20] and dynamic resource allocation [21]. However, a common limitation across these works is their treatment of the physical network as a monolithic entity. They struggle to meet the diverse and customized needs of different users simultaneously. Reference [22] analyzed how dynamic network slice allocation delays affect latency, bandwidth, and service continuity, showing that reducing allocation time enhances performance for mobile users in 5G networks. While RAN slicing is a promising solution to this problem, its application in the complex SAGIN context remains strikingly underexplored, creating a clear research opportunity.

In the domain of UAV trajectory optimization, the focus has been on leveraging UAVs’ flexibility and LoS links to improve performance. Works like [23] jointly optimized trajectory and power control, and [24,25] aimed to minimize delay through integrated trajectory and resource planning. Ref. [26] applied the Successive Convex Approximation (SCA) method to solve the subproblems of computation resource and bandwidth allocation as well as UAV trajectory optimization. Notwithstanding these contributions, the vast majority of these studies operate in idealized environments. A critical oversight is the prevalent neglect of No-Fly Zone (NFZ) constraints, which are non-negotiable for real-world UAV deployment around airports, power lines, or military zones. Consequently, many proposed trajectories are academically interesting but practically infeasible, highlighting the need for trajectory designs that explicitly incorporate such safety-critical constraints.

On the front of user association and slice resource allocation, References [27,28] investigated the user association problem using evolutionary game theory and the Lagrangian dual method, respectively. The joint optimization of user association and resource allocation has been extensively studied in [29,30,31]. Ref. [29] used a D3QN-based method for resource allocation, and [31] developed a MADDPG framework for joint user association and allocation. Ref. [32] proposed a fractional programming approach using quadratic transform, weighted MMSE, and SCA for beamforming, offloading, and resource allocation. Ref. [33] proposed an energy-efficient user selection algorithm with dynamic caching. In addition, ref. [34] introduced a multi-agent deep reinforcement learning-based strategy for resource allocation in vehicular networks. Despite their sophistication, these approaches are often myopic. They predominantly focus on single-dimension optimization within a static or ground-based network topology. Crucially, they fail to account for the dynamic network topology introduced by mobile UAVs and the intricate coupling between association decisions, resource slicing policies, and UAV trajectories. Furthermore, many DRL solutions rely on algorithms like DQN, DDPG, or TD3, which are known to suffer from training instability and inefficiency in high-dimensional continuous action spaces, precisely the nature of the resource allocation problem here.

Therefore, in contrast to the existing literature, our work proposes a comprehensive solution that bridges these gaps. We introduce a joint optimization framework that (1) incorporates RAN slicing into SAGIN for customized service provisioning, (2) rigorously embeds NFZ constraints into UAV trajectory planning to ensure practicality, and (3) simultaneously solves user association, slice resource allocation, and trajectory optimization. We employ the Soft Actor–Critic (SAC) algorithm, which is specifically designed for stability and efficiency in high-dimensional continuous action spaces, thereby addressing the limitations of prior DRL approaches used in this domain.

To clearly highlight the differences between this study and existing literature and emphasize its novelty, we compared it with previous works, as shown in Table 3.

## 3. System Model

### 3.1. Network Model

**Figure 1:**
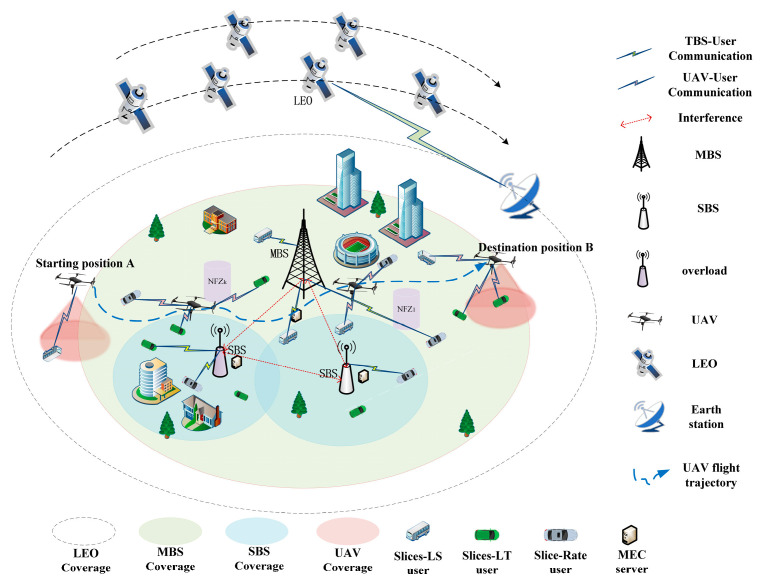
System scenario of network slicing in a space–air–ground integrated network.

As shown in Figure 1, we study a SAGIN consisting of B terrestrial base stations (TBSs, including macro base stations (MBSs) and small base stations (SBSs)), I ground users, U UAVs, and a constellation of low earth orbit (LEO) satellites. The coverage area of each MBS contains multiple SBSs. UAVs act as aerial base stations and fly from a starting point $l_{0}$ to a destination point $l_{e}$ at a constant power to provide services to ground users. The UAV service area is denoted as the region $x_{\max}\;\times \;y_{\max}$, within which several no-fly zones (NFZs) are defined to prohibit UAV traversal. Due to the limited coverage of terrestrial base stations and their potential damage to these stations in disaster areas, which can disrupt communications, UAVs and LEO satellites are introduced to achieve seamless coverage across the entire cell. This ensures continuous communication services for ground users. Each user can establish a communication link with terrestrial base stations, UAVs, or LEO satellites. We assume that sets $W\;=\;\{W_{0},\ldots ,\;W_{B+U}\}$ and $F\;=\;\{F_{0},\ldots ,\;F_{B+U}\}$ represent the total virtual bandwidth and computing resources of all base stations, respectively. The bandwidth and computing resources of each terrestrial base station and UAV are partitioned into three service slices: SLS, SLT, and SR, which correspond to latency-sensitive, latency-tolerant, and high-data-rate tasks, respectively. Accordingly, the set of all slices is denoted by n∈N = {SLS, SLT, SR}. This resource slicing scheme can be applied to any number of service slices [35].

In addition, we define the sets of LEO satellites, TBSs, and UAVs as $L\;=\;\left\{1,\;2,\ldots ,\;L\right\},\;B\;=\;\left\{1,\;2,\ldots ,\;B\right\}$, and U = {1, 2,…, U}, respectively. The set of all base stations is denoted as J = L∪B∪U. The set of users associated with slice n is denoted as $I_{n}\;=\;\{1,\ldots ,\;M_{1},\;\ldots ,\;M_{1}\;+\;M_{2},\ldots ,\;M_{1}\;+\;M_{2}\;+\;M_{3}\}$, while $I_{M_{1}}\;=\;\{1,\;2,\ldots ,\;M_{1}\},\;I_{M_{2}}\;=\;\{1,\;2,\ldots ,\;M_{2}\}$, and $I_{M_{3}}\;=\;\{1,\;2,\ldots ,\;M_{3}\}$ represent the sets of users served by the SLS, SLT, and SR slices, respectively. We also define a binary association variable $x_{n_{i},j}\left(t\right)$ to indicate whether user i on slice n is associated with base station j, where $x_{n_{i},j}\left(t\right)\;=\;1$ if associated, otherwise, $x_{n_{i},j}\left(t\right)\;=\;0$. At each time slot t, each user i on slice n can be associated with only one base station, i.e., $\sum_{j\in J}x_{n_{i},j}\left(t\right)\;=\;1$. The bandwidth and computing resources allocated by the jth base station to slice n are denoted by $W_{n}\;=\;b_{n,j}\left(t\right)W_{j}$ and $F_{n}\;=\;f_{n,j}\left(t\right)F_{j}$, respectively. $b_{n,j}\left(t\right)$ and $f_{n,j}\left(t\right)$ represent the allocation ratios of bandwidth and computing resources assigned to slice n, respectively, and $b_{n,j}\left(t\right)\in \left[0,\;1\right],\;f_{n,j}\left(t\right)\in \left[0,\;1\right]$.

### 3.2. UAV Mobility Model

To simplify the modeling, we assume that all UAVs fly at a fixed altitude H. Based on the 3D Cartesian coordinate system, the position of the uth UAV at time slot t is denoted by $l_{u}(t)\;=\;\left\{x_{u}^{UAV}\left(t\right),\;y_{u}^{UAV}\left(t\right),\;H\right\}$, and its projection onto the horizontal plane is denoted by $l_{u}^{\prime }(t)\;=\;\left\{x_{u}^{UAV}\left(t\right),\;y_{u}^{UAV}\left(t\right)\right\}$. The UAV mobility is subject to a set of constraints, including its initial and final positions, which are expressed as follows.

(1) $$l_{u}^{\prime }(0)=l_{0},l_{u}^{\prime }(T)=l_{e}$$

In addition, the UAV’s flight speed is denoted as $V_{u}\left(t\right)\;=\;\frac{l_{u}^{\prime }(t\;+\;1)\;-\;l_{u}^{\prime }(t)}{\tau }$ [36]. Furthermore, considering the constraints of the service area, as well as the UAV’s minimum and maximum speed requirements, denoted by $V_{\min}$ and $V_{\max}$, respectively, the UAV’s trajectory and flight speed should satisfy the following conditions.

(2) $$0\leq x_{u}^{UAV}\left(t\right)\leq x_{\max}$$

(3) $$0\leq y_{u}^{UAV}\left(t\right)\leq y_{\max}$$

(4) $$\;V_{\min}^{2}\leq {\|V_{u}(t)\|}^{2}\leq V_{\max}^{2}$$

Due to limitations in horizontal flight speed, the UAV’s flight distance is also constrained and must satisfy the following condition.

(5) $$\Delta l_{u}^{\prime }(t)=\|l_{u}^{\prime }(t+1)-l_{u}^{\prime }(t)\|\leq L_{\max}^{x}$$

where $\Delta l_{u}^{\prime }(t)$ denotes the UAV’s horizontal travel distance, and $L_{\max}^{x}$ represents the maximum allowable horizontal distance.

### 3.3. NFZ Model

**Figure 2:**
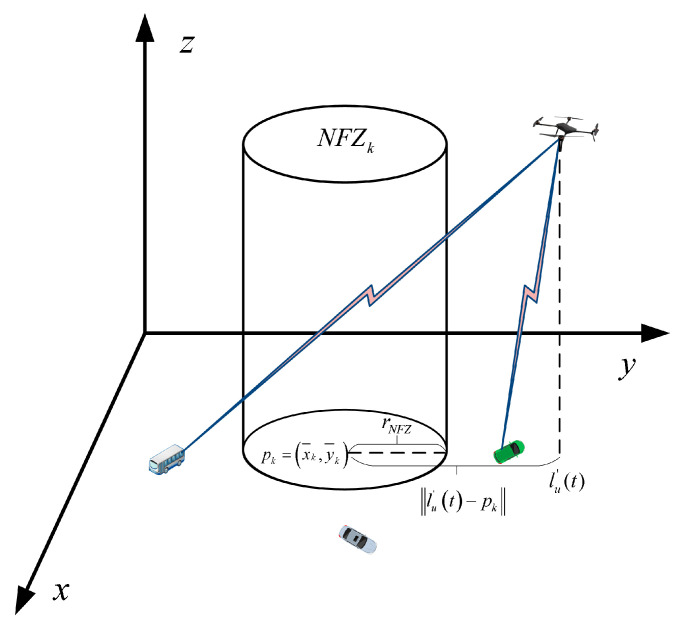
Communication system with No-Fly Zones.

In certain specific areas, there are K randomly distributed but non-overlapping NFZs that prohibit UAVs from flying through [37], we model each NFZ as a cylinder of infinite height [14], which is a common simplification for conservative trajectory planning. This implies that a UAV violates the NFZ constraint if its horizontal projection enters the circular area of the NFZ, regardless of its altitude. Moreover, we consider NFZs with a radius of $r_{NFZ}$ and a sufficiently high altitude. The set of NFZs is denoted by $K\;=\;\left\{1,\;2,\ldots ,\;K\right\}$, and the center of the kth NFZ is represented by $p_{k}\;=\;\left({\bar{x}}_{k},\;{\bar{y}}_{k}\right)$, as shown in Figure 2. The NFZ constraint is formulated solely based on the horizontal distance.

(6) $${\|l_{u}^{\prime }\left(t\right)-p_{k}\|}^{2}\geq r_{NFZ}^{2},\forall u\in U,k\in K$$

The radius $r_{NFZ}$ is usually set proportionally to the size of the simulation scenario, reflecting a typical safety margin around sensitive areas. The assumption of infinite height ($H_{NFZ}\;\to \;\infty$) is valid as long as the UAV’s operational altitude H is significantly lower than any realistic NFZ ceiling, which holds true for our scenario.

### 3.4. Communication Model

In wireless communication, the distance between the sender and the receiver is an important factor to consider. For convenience, we assume the geographical position of the LEO satellite is $l_{l}\left(t\right)\;=\;\left\{x_{l}^{LEO}\left(t\right),\;y_{l}^{LEO}\left(t\right),\;z_{l}^{LEO}\left(t\right)\right\}$, the location of the bth TBS is defined as $l_{b}\left(t\right)\;=\;\left\{x_{b}^{TBS}\left(t\right),\;y_{b}^{TBS}\left(t\right),\;z_{b}^{TBS}\left(t\right)\right\}$, and the coordinates of user i on slice n are given by $l_{n_{i}}\left(t\right)=\left\{x_{n_{i}}\left(t\right),y_{n_{i}}\left(t\right),0\right\}$. Considering a 3D Cartesian coordinate system, at any time t, the projected positions of LEO l, TBS b, and user i on the horizontal plane are denoted by $l_{l}^{\prime }\left(t\right)=\left\{x_{l}^{LEO}\left(t\right),\;y_{l}^{LEO}\left(t\right)\right\},l_{b}^{\prime }\left(t\right)=\left\{x_{b}^{TBS}\left(t\right),y_{b}^{TBS}\left(t\right)\right\}$, and $l_{n_{i}}^{\prime }\left(t\right)=\left\{x_{n_{i}}\left(t\right),y_{n_{i}}\left(t\right)\right\}$, respectively. Accordingly, the distances between LEO l, TBS b, UAV u and user i on slice n are denoted as $d_{n_{i},l}\left(t\right)=\sqrt{{\left(z_{l}^{LEO}(t)\right)}^{2}+{\left\|l_{l}^{\prime }\left(t\right)-l_{n_{i}}^{\prime }\left(t\right)\right\|}^{2}}$, $d_{n_{i},b}\left(t\right)=\sqrt{{\left(z_{b}^{TBS}(t)\right)}^{2}+{\left\|l_{b}^{\prime }\left(t\right)-l_{n_{i}}^{\prime }\left(t\right)\right\|}^{2}}$ and $d_{n_{i},u}\left(t\right)=\sqrt{H^{2}+{\left\|l_{u}^{\prime }(t)-l_{n_{i}}^{\prime }\left(t\right)\right\|}^{2}}$, respectively.

#### 3.4.1. LEO-User Communication Model

With the aid of LEO satellite downlinks, seamless coverage can be provided to users. Since the distance between the satellite and the user is relatively large, the impact of user mobility on the channel gain can be neglected. The channel coefficient between user i on slice n and LEO l is denoted as

(7) $$g_{n_{i},l}^{down}\left(t\right)=h_{ni,l}\left(t\right){\left[d_{ni,l}\left(t\right)\right]}^{-\alpha }={\left(\frac{c}{4\pi f_{c}}\right)}^{2}{\left[d_{ni,l}\left(t\right)\right]}^{-\alpha }$$

where $h_{n_{i},l}\left(t\right)$ denotes the unit radio propagation loss of the satellite link due to free-space path loss [12]. c represents the speed of light, $f_{c}$ denotes the carrier frequency, $d_{n_{i},l}\left(t\right)$ is the distance between LEO l and user i, and α is the path loss exponent. Therefore, at time slot t, the downlink transmission rate of user i on slice n associated with LEO l is

(8) $$r_{n_{i},l}^{down}\left(t\right)=x_{ni,l}\left(t\right)y_{l,ni}\left(t\right)W_{l}\log\left(1+\frac{p_{l,n_{i}}^{down}\left(t\right)g_{n_{i},l}^{down}\left(t\right)}{\sigma^{2}}\right)$$

where the transmission power of LEO l to user i on slice n is denoted by $p_{l,n_{i}}^{down}\left(t\right)$, and $x_{n_{i},l}\left(t\right)$ is a binary variable indicating whether user i is associated with LEO l. $\sigma^{2}$ represents the noise variance, and $y_{l,ni}\left(t\right)$ denotes the proportion of bandwidth resources allocated to user i on slice n, associated with LEO l.

For users on both latency-tolerant and latency-sensitive slices, computing tasks need to be offloaded to MEC servers for processing. Accordingly, the uplink data transmission rate of user i on slice n when offloading tasks to the MEC server deployed on LEO satellite l is

(9) $$r_{n_{i},l}^{up}\left(t\right)=x_{ni,l}\left(t\right)y_{l,ni}\left(t\right)W_{l}\log\left(1+\frac{p_{n_{i},l}^{up}\left(t\right)g_{n_{i},l}^{up}\left(t\right)}{\sigma^{2}}\right)$$

#### 3.4.2. UAV–User Communication Model

In the UAV–user communication model, the UAV acts as an aerial base station providing different types of services to users via the downlink. Similarly, at time slot t, the channel gain between UAV u and user i on slice n can be expressed as

(10) $$g_{n_{i},u}^{down}\left(t\right)=h_{ni,u}\left(t\right){\left[d_{ni,u}\left(t\right)\right]}^{-\alpha }$$

It is assumed that the small-scale fading component $h_{n_{i},u}\left(t\right)$ follows a Rayleigh fading channel model and is given by $h_{n_{i},u}\left(t\right)=h_{0}\left[\frac{R}{R+1}{\hat{h}}_{n_{i},u}\left(t\right)+\frac{1}{R+1}{\tilde{h}}_{n_{i},u}\left(t\right)\right]$, where $h_{0}$ denotes the reference channel gain at a distance of 1 m, R represents the Rician factor, ${\hat{h}}_{n_{i},u}\left(t\right)$ is the Line-of-Sight (LoS) component, ${\tilde{h}}_{n_{i},u}\left(t\right)$ is the Non-Line-of-Sight (NLoS) component, and ${\tilde{h}}_{n_{i},u}\left(t\right)\sim CN(0,\;1)$. Accordingly, the data transmission rate between UAV u and user i on slice n at time slot t can be expressed as follows.

(11) $$r_{n_{i},u}^{\mathrm{down}}(t)=x_{n_{i},u}(t)\cdot y_{u,n_{i}}(t)\cdot W_{u}\log_{2}\left(1+\frac{p_{u,n_{i}}^{\mathrm{down}}(t)\cdot g_{n_{i},u}^{\mathrm{down}}(t)}{\sum_{u^{\prime }\in U\setminus \{u\}}p_{u^{\prime },n_{i}}^{\mathrm{down}}(t)\cdot g_{n_{i},u^{\prime }}^{\mathrm{down}}(t)+\sigma^{2}}\right)$$

Similarly, the uplink data transmission rate for user i offloading tasks to the MEC server equipped on UAV u is

(12) $$r_{n_{i},u}^{\mathrm{up}}(t)=x_{n_{i},u}(t)\cdot y_{u,n_{i}}(t)\cdot W_{u}\cdot \log\left(1+\frac{p_{n_{i},u}^{\mathrm{up}}(t)\cdot g_{n_{i},u}^{\mathrm{up}}(t)}{\sum_{u^{\prime }\in U\backslash \{u\}}p_{n_{i},u^{\prime }}^{\mathrm{up}}(t)\cdot g_{n_{i},u^{\prime }}^{\mathrm{up}}(t)+\sigma^{2}}\right)$$

#### 3.4.3. TBS-User Communication Model

In the communication model between TBSs and users, each TBS provides customized services to multiple users. At time slot t, the channel gain between user i and TBS b is defined as $g_{n_{i},b}^{down}\left(t\right)=h_{n_{i},b}\left(t\right){\left[d_{n_{i},b}\left(t\right)\right]}^{-\alpha }$, where $h_{n_{i},b}\left(t\right)$ follows an exponential distribution with unit mean [5]. Therefore, at time slot t, the downlink data transmission rate between terrestrial base station b and user i on slice n is

(13) $$r_{n_{i},b}^{\mathrm{down}}(t)=x_{n_{i},b}(t)\cdot y_{b,n_{i}}(t)\cdot W_{b}\cdot \log\left(1+\frac{p_{b,n_{i}}^{\mathrm{down}}(t)\cdot g_{n_{i},b}^{\mathrm{down}}(t)}{\sum_{b^{\prime }\in B\setminus \{b\}}p_{b^{\prime },n_{i}}^{\mathrm{down}}(t)\cdot g_{n_{i},b^{\prime }}^{\mathrm{down}}(t)+\sigma^{2}}\right)$$

Similarly to the satellite and UAV cases, when user i offloads a task to the MEC server equipped at terrestrial base station b, the uplink data transmission rate is

(14) $$r_{n_{i},u}^{\mathrm{up}}(t)=x_{n_{i},u}(t)\cdot y_{u,n_{i}}(t)\cdot W_{u}\cdot \log\left(1+\frac{p_{n_{i},u}^{\mathrm{up}}(t)\cdot g_{n_{i},u}^{\mathrm{up}}(t)}{\sum_{u^{\prime }\in U\backslash \{u\}}p_{n_{i},u^{\prime }}^{\mathrm{up}}(t)\cdot g_{n_{i},u^{\prime }}^{\mathrm{up}}(t)+\sigma^{2}}\right)$$

#### 3.4.4. Computation Model

Each user i is required to process a computation task at each time slot t, which can be represented by a tuple $\Omega_{n_{i}}\left(t\right)=\left\{L_{n_{i}}\left(t\right),C_{n_{i}}\left(t\right),\varphi_{n}^{comp}\right\}$. $L_{n_{i}}\left(t\right)$ denotes the data size, $C_{n_{i}}\left(t\right)$ is the required number of CPU cycles per bit, and $\varphi_{n}^{comp}$ represents the maximum tolerable delay threshold allowed for slice n. Considering that the amount of data generated by computation is relatively small compared to the total task data, the download time can be neglected. Therefore, the total service delay experienced by the user mainly consists of two components: transmission delay and computation delay. The transmission delay can be expressed as

(15) $$T_{n_{i},j}^{up}\left(t\right)=\frac{L_{n_{i}}\left(t\right)}{r_{n_{i},j}^{up}\left(t\right)}$$

Let $f_{n_{i},j}\left(t\right)$ (CPU cycles per second) denote the amount of computing resources allocated to user i on slice n. Then, the computation delay for processing the task on slice n can be expressed as

(16) $$T_{n_{i},j}^{com}\left(t\right)=\frac{C_{n_{i}}\left(t\right)}{f_{n_{i},j}\left(t\right)}$$

Therefore, for user i associated with base station j, the total delay can be expressed as

(17) $$T_{n_{i},j}\left(t\right)=T_{n_{i},j}^{up}\left(t\right)+T_{n_{i},j}^{com}\left(t\right)$$

In the above expression, the two terms represent the transmission delay and the processing delay of the task, respectively. Moreover, the computational capabilities vary across different types of base stations. The SLT slice is designed for applications that can tolerate a certain degree of delay but have requirements for high reliability and substantial data volume. Therefore, for a user ii associated with the SLT slice, the constraint is defined by a maximum tolerable latency threshold $\varphi_{\mathrm{SLT}}^{comp}$, The constraint is formalized as:

(18) $$T_{n,j}\left(t\right)\leq \varphi_{\mathrm{SLT}}^{comp},\;\forall n=\mathrm{SLT}$$

For users in the SLS slice, which caters to ultra-reliable low-latency communication (URLLC) services such as autonomous driving and industrial control, the QoS requirement is the most stringent. The total service delay must not exceed a very small maximum tolerable latency threshold, and the service must be highly reliable. Therefore, the QoS constraint for a user ii associated with the SLS slice is defined as follows:

(19) $$T_{n,j}\left(t\right)\leq \varphi_{\mathrm{SLS}}^{comp},\;\forall n=\mathrm{SLS}$$

where $\varphi_{\mathrm{SLS}}^{comp}$ is the maximum end-to-end latency threshold for the SLS slice, and its value is typically much smaller than that of the SLT slice (i.e., $\varphi_{\mathrm{SLS}}^{comp}\leq \varphi_{\mathrm{SLT}}^{comp}$).

For the SR slice, the primary concern is the transmission rate of users. Therefore, the transmission rate of a user assigned to the SR slice should exceed the minimum rate threshold defined for the slice, i.e.,

(20) $$r_{n_{i},j}\left(t\right)⩾R_{e}$$

## 4. Problem Formulation

In this section, our objective is to minimize the overall system cost while maximizing system utility, subject to the service requirements of users across different network slices.

### 4.1. System Cost

The total system cost of network slicing consists of the operational cost and the reconfiguration cost of the slices.

#### 4.1.1. Operational Cost

The operational cost of a slice depends on the bandwidth and computational resources allocated to it by the base stations. Thus, at time slot t, the operational cost can be expressed as

(21) $$U_{o}(t)=\sum_{n\in N}\sum_{i\in I_{n}}\sum_{j\in J}\left(\zeta_{o,j,b}b_{n,j}(t)W_{j}+\zeta_{o,j,f}f_{n,j}(t)F_{j}\right)$$

where $\zeta_{o,j,b}$ and $\zeta_{o,j,f}$ denote the unit costs of bandwidth and computational resources at base station j, respectively.

#### 4.1.2. Reconfiguration Cost

At different time slots t, the service requests of user i may vary dynamically, necessitating adjustments in resource allocation strategies. Moreover, the reconfiguration cost of a slice is closely related to the type of user service. The reconfiguration cost at time slot t is defined as follows

(22) $$U_{r}(t)=\sum_{n\in N}\sum_{i\in I_{n}}\sum_{j\in J}\left(\zeta_{r,j,b}\cdot C_{1}+\zeta_{r,j,f}\cdot C_{2}\right)$$

For users in the SR slice:

(23) $$C_{1}={\left[b_{n,j}\left(t\right)W_{j}-b_{n,j}\left(t-1\right)W_{j}\right]}^{+}$$

(24) $$C_{1}=\left\{\begin{array}{ll} b_{n,j}(t)W_{j}-b_{n,j}(t-1)W_{j}, & \mathrm{if}\;b_{n,j}(t)W_{j}>b_{n,j}(t-1)W_{j}\\ 0, & \mathrm{otherwise} \end{array}\right.$$

For users in computation offloading slices:

(25) $$C_{2}={\left[f_{n,j}\left(t\right)F_{j}-f_{n,j}\left(t-1\right)F_{j}\right]}^{+}$$

(26) $$C_{2}=\left\{\begin{array}{ll} f_{n,j}\left(t\right)F_{j}-f_{n,j}\left(t-1\right)F_{j}, & \;\mathrm{if}\;f_{n,j}\left(t\right)F_{j}>f_{n,j}\left(t-1\right)F_{j}\\ 0 & \;\mathrm{otherwise} \end{array}\right.$$

Based on the above analysis, the total system cost of network slicing can be expressed as follows

(27) $$U_{\mathrm{cost}}\left(t\right)=U_{o}\left(t\right)+U_{r}\left(t\right)$$

### 4.2. System Revenue

In this paper, the system revenue is defined as consisting of two components: the communication rate utility and the computational efficiency utility. Therefore, the total system revenue can be expressed as follows

(28) $$U_{\mathrm{renu}}(t)=\sum_{n\in N}\sum_{i\in I_{n}}\sum_{j\in J}\omega_{1}r_{n_{i},j}(t)+\omega_{2}\frac{C_{n_{i}}(t)}{T_{n_{i},j}^{\mathrm{com}}(t)}$$

The joint problem of user association, slice resource allocation, and UAV trajectory optimization is formulated with the objective of maximizing the overall system utility while minimizing the total system cost. Therefore, the system optimization objective is defined as follows

(29) $$P1:\underset{x,b,f,l}{\max}\sum_{t\in T}\left(U_{\mathrm{renu}}(t)-U_{\mathrm{cost}}(t)\right)$$

(29a) $$C1:0\leq x_{u}^{\mathrm{UAV}}(t)\leq x_{\max},\forall u\in U$$

(29b) $$C2:0\leq y_{u}^{UAV}(t)\leq y_{\max},\forall u\in U$$

(29c) $$C3:\;V_{\min}^{2}\leq {\left\|V_{u}(t)\right\|}^{2}\leq V_{\max}^{2},\forall u\in U$$

(29d) $$C4:\Delta l_{u}^{\prime }(t)=\|l_{u}^{\prime }(t+1)-l_{u}^{\prime }(t)\|\leq L_{\max}^{x},\forall u\in U$$

(29e) $$C5:\|l_{u}^{\prime }(t)-p_{k}{\|}^{2}\geq r_{NFZ}^{2},\forall u\in U,k\in K$$

(29f) $$C6:T_{n_{i},j}(t)\leq \varphi_{n}^{\mathrm{comp}},n\in \left\{SLS,SLT\right\},i\in I_{n},j\in J$$

(29g) $$C7:r_{n_{i},j}\left(t\right)\geq R_{e},\forall n\in N,i\in I,j\in J$$

(29h) $$C8:\;b_{n,j}\left(t\right)\in [0,1],\forall n\in N,j\in J$$

(29i) $$C9:\;f_{n,j}\left(t\right)\in [0,1],\forall n\in N,j\in J$$

(29j) $$C10:\;\sum_{n\in N}b_{n,j}\left(t\right)=1,\forall n\in N,$$

(29k) $$C11:\sum_{n\in N}f_{n,j}\left(t\right)=1,\forall n\in N$$

(29l) $$C12:x_{n_{i},j}\left(t\right)\in \left\{0,1\right\},\;\forall n\in N,i\in I_{n},j\in J$$

(29m) $$C13:\sum_{j\in J}x_{n_{i},j}\left(t\right)\leq 1,\forall n\in N,i\in I_{n}$$

(29n) $$C14:\sum_{n\in N}B_{n}\leq W_{j},\;\forall j\in J$$

(29o) $$C15:\;\sum_{n\in N}F_{n}\leq F_{j},\;\forall j\in J$$

where $x=\left\{x_{n_{i},j}\left(t\right)\right\}$ denotes the set of user-associated variables, while $b=\left\{b_{n,j}\left(t\right)\right\}$ and $f=\left\{f_{n,j}\left(t\right)\right\}$ represents the set of bandwidth resource allocation ratios and the set of computing resource allocation ratios. The $l=\left\{l_{u}(t)\right\}$ denotes the set of UAV trajectories. Constraints C1–C4 define the limitations on UAV flight trajectory and velocity; C5 represents the NFZ constraint; C6–C7 impose requirements on the data transmission rate and service delay for slice n; C8–C9 specify the proportions of bandwidth and computing resources that base station j allocates to user i on slice n; C10–C11 indicate that the total available bandwidth and computing resources of the base station are distributed among all users; C12 defines the user association as a binary variable, when $x_{n_{i},j}\left(t\right)\;=\;1$, it implies that user i on slice n is associated with base station j, otherwise $x_{n_{i},j}\left(t\right)\;=\;0$; C13 ensures that each user can connect to at most one base station; and C14 and C15 represent the total inter-slice constraints on bandwidth and computing resources, respectively.

**Figure 3:**
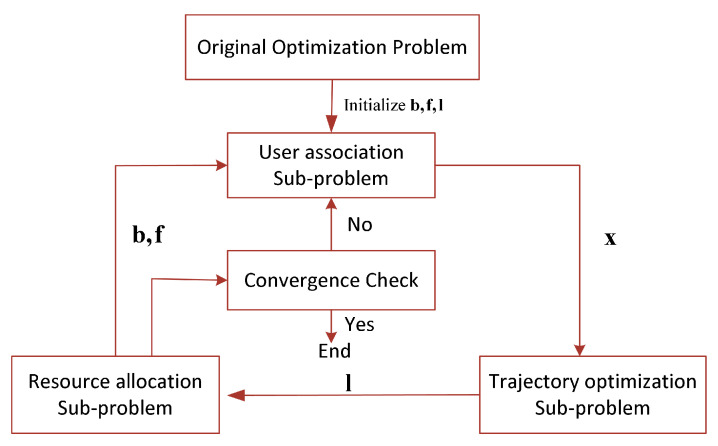
Flowchart of the Proposed Algorithm.

Solving the original optimization problem is challenging for the following reasons. First, the convexity of the objective function is not guaranteed. Second, the user association variable $x_{n_{i},j}\left(t\right)$ is binary, which introduces integer constraints, and constraint C12 is non-convex. Therefore, the objective function forms a mixed-integer non-convex optimization problem, which is generally difficult to solve optimally. To address the computational complexity brought by the mixed-integer non-convex nature of the original problem, we relax the binary user association variables and further decompose the problem into three subproblems: user association, resource allocation, and UAV trajectory optimization, as illustrated in Figure 3.

These three subproblems are not solved by a simple one-time sequential process but are instead iteratively optimized within an alternating optimization framework, as illustrated in Figure 3. The workflow of the proposed SAC-MS algorithm is as follows: in each iteration, the UA subproblem is first solved while keeping the resource allocation and trajectory fixed. Then, using the updated association strategy and resource allocation scheme, the TO subproblem is solved. Finally, the resource allocation subproblem is addressed based on the updated association strategy and trajectory. This alternating process is repeated until convergence.

Specifically, given the bandwidth allocation ratio $b_{n,j}\left(t\right)$, the computation resource allocation ratio $f_{n,j}\left(t\right)$, and the UAV trajectory optimization strategy $l_{u}\left(t\right)$, the user association problem can be formulated as follows

(30) $$sub-UA:\underset{x}{\max}\sum_{t\in T}\left(U_{\mathrm{renu}}(t)-U_{\mathrm{cost}}(t)\right)$$

(30a) s.t. C6–C7, C12–C13

Given the bandwidth allocation $b_{n,j}\left(t\right)$, computing resource allocation $f_{n,j}\left(t\right)$, and user association strategy $x_{n_{i},j}\left(t\right)$ in **P1**, the UAV positions can be optimized by solving the following problem.

(31) $$sub-TO:\underset{l}{\max}\sum_{t\in T}\sum_{n\in N}\sum_{i\in I_{n}}\sum_{j\in J}\omega_{1}r_{n_{i},j}(t)$$

(31a) s.t. C1–C7

Given the user association strategy $x_{n_{i},j}\left(t\right)$ and the UAV position $l_{u}\left(t\right)$, the problem of bandwidth and computation resource allocation can be formulated as follows

(32) $$sub-RA:\underset{b,f}{\max}\sum_{t\in T}\left(U_{\mathrm{renu}}(t)-U_{\cos t}(t)\right)$$

(32a) s.t. C6–C11, C14–C15

## 5. Proposed Algorithm

In this section, we propose a joint user association, UAV trajectory optimization, and resource allocation algorithm (SAC-MS) to iteratively solve the three subproblems mentioned above. First, a matching game approach is adopted for the user association problem. For the UAV trajectory optimization problem, auxiliary variables are introduced, and the SCA method is employed to solve it. Finally, the resource allocation problem is addressed using a reinforcement learning algorithm to learn the optimal strategy for bandwidth and computation resource allocation.

### 5.1. User Association (UA)

**Figure 4:**
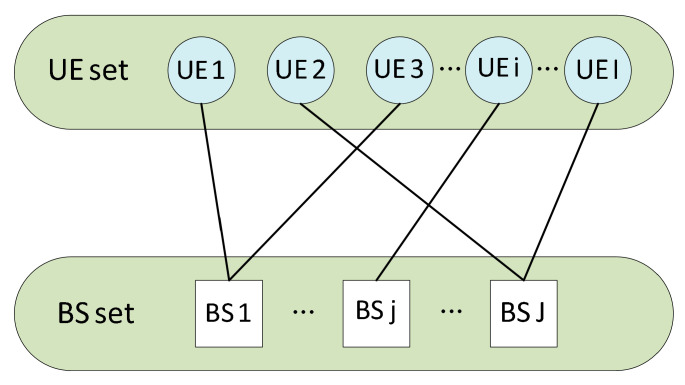
Many-to-one matching game between users and BSs.

To solve the user association subproblem with reduced computing complexity, we apply many-to-one matching theory to obtain the optimal association strategy. In this model, each user can associate with only one base station, while each base station can serve multiple users. Therefore, as illustrated in Figure 4, the relationship between users and base stations is modeled as a many-to-one matching problem.

Formally, the user association problem can be modeled as a many-to-one matching game, represented by a tuple $J,\;I,\;{≻}_{J},\;{≻}_{I}$, where ${≻}_{J}={\left\{{≻}_{j}\right\}}_{j\in J},\;{≻}_{I}={\left\{{≻}_{i}\right\}}_{i\in I}$ denote the preference sets of base stations and users, respectively. The user association matching game is denoted by θ, and the many-to-one matching between the base station set J and the user set I must satisfy the following conditions [38].

-
$\theta \left(i\right)\subset J,\left|\theta (i)\right|⩽1,\forall i\in I_{n}$

-
$\theta (j)\subset I_{n},\left|\theta \left(j\right)\right|⩽I_{j}^{\max},\forall i\in I_{n}$

-
$\theta \left(i\right)=j\Leftrightarrow i\in \theta \left(j\right)$

The first property restricts each user to be paired with only one base station. The second property limits the number of users that can be matched with each base station. The third property requires that a user can only be associated with a base station if the base station agrees to provide service to that user.

Let $S_{j}\left(i\right)$ and $S_{i}\left(j\right)$ denote the utility functions of base station j for user i, and of user i for base station j, respectively. If user i prefers base station j over base station $j^{\prime }$, i.e., $S_{i}(j)>S_{i}(j^{\prime })$, where $j,\;j^{\prime }\in J$, this preference relationship is denoted as $j{≻}_{i}j^{\prime }$. Conversely, if base station j prefers user i over user $i^{\prime }$, i.e., $S_{j}(i)>S_{j}(i^{\prime })$, where $i,\;i^{\prime }\in I$, this preference relationship is denoted as $i{≻}_{j}i^{\prime }$ [39].

Utility Function of Users: Considering resource efficiency, each user tends to select the base station that can allocate more resources. Based on this, the user’s utility function is modeled as the throughput per unit of energy consumption, i.e.,

(33) $$S_{i}\left(j\right)=\frac{x_{n_{i},j}\left(t\right)r_{n_{i},j}\left(t\right)}{p_{n_{i},j}\left(t\right)}$$

Utility Function of Base Stations: To enhance overall system performance, each base station tends to serve users with the strongest received signal strength. Accordingly, the utility function of a base station is defined based on the signal quality of its associated users, aiming to maximize system utility, i.e.,

(34) $$S_{j}\left(i\right)=p_{n_{i},j}\left(t\right)g_{n_{i},j}\left(t\right)$$

The user utility function and the base station utility function are not independent optimization objectives, but preference indicators in the many-to-one matching game. Although stable matching does not strictly maximize system utility, it eliminates all blocking pairs and achieves a Pareto-efficient equilibrium, thus effectively enhancing system performance. We provide the pseudocode for addressing the user association problem using a many-to-one bilateral stable matching game, as shown in Algorithm 1, and analyzed the computational complexity of the algorithm.

We provide the pseudocode for addressing the user association problem using a many-to-one bilateral stable matching game, as shown in Algorithm 1, and analyze the computational complexity of the algorithm.

**Algorithm 1:** Association Algorithm Based on Stable Matching Game.1:Input: Preference matrices for BSs and users, Utility functions for both BSs and users to calculate preferences2:Ouput: Stable matching results between BSs and users3:Initialize: 4:  (a) Set all users as “free” (not yet matched);5:  (b) Set all BSs with empty connected user lists;6:  (c) Set BSs’ rejection list;7:  (d) Set a list of requests for each user;8:Composition of preference lists:9:  (a) BS and user exchange their listing information;10:  (b) Each user constructs its preference list based on its own Utility Function $S_{i}\left(j\right)=\frac{x_{n_{i,}j}\left(t\right)r_{n_{i},j}\left(t\right)}{p_{n_{i},j}\left(t\right)}$, and rank BSs in descending order of preference;11:  (c) Each BS constructs its preference list based on its own Utility Function  $S_{j}\left(i\right)=p_{n_{i},j}\left(t\right)g_{n_{i},j}\left(t\right)$, and rank users in descending order of preference;12:Match Process:13:Repeat:14:  **For** each user i∈I that is free:15:    i applies to the highest-ranked BS j in its preference list;16:  **For** each BS j∈J:17:   (a) j receives applications from users;18:   (b) Sort users in the application list according to BS j preference list;19:   **If** the number of applicants for j exceeds the quota:20:     (a) Select the top $I_{n}$ users based on preference;21:     (b) Reject the remaining users and add them to j rejection list;22:   **else:**23:     (a) Accept all applicants and add them to BS j waiting list;24:     (b) Add accepted UAVs to n’s matched UAVs list;25: **For** each user i∈I:26:  **If** i is rejected by a BS:27:    (a) i re-applies to the next most preferred BS;28:    (b) Update the application list of i and write down the BS that has been applied;29:  **end if**30: **end for**31: **For** each BS j∈J:32:    (a) Update the waiting list based on the newly accepted users;33:    (b) Combine the original waiting list with the newly accepted users;34: **end for**

The computational complexity of the user association matching game consists of the complexity of constructing the participants’ preference lists and the complexity of executing the game. Each user ranks the base stations in descending order according to its own preferences, thereby generating its preference list. This ranking step results in a computational complexity of $O\left(J\log\left(J\right)\right)$ for each user, and thus $O\left(JI\log\left(J\right)\right)$ for I users. Similarly, the computational complexity for J base stations is $O\left(JI\log\left(I\right)\right)$. Therefore, the total complexity of constructing preference lists for all users and base stations is $O\left(JI\log\left(JI\right)\right)$. During the execution of the game, each user sends an association request to a base station, and the iteration coefficient of the game determines its execution complexity. Accordingly, the computational complexity of executing the game is $O\left(I\right)$. However, the complexity of constructing the preference lists is more significant than that of executing the game. Hence, the overall computational complexity of Algorithm 1 is $O\left(JI\log\left(JI\right)\right)$.

### 5.2. UAV Trajectory Optimization (TO)

After obtaining the user association strategy $x_{n_{i},j}\left(t\right)$, we incorporate it into the UAV trajectory optimization subproblem, which results in the following formulation.

(35) $$\begin{array}{l} \mathrm{sub}-\mathrm{TO}:\underset{l}{\max}\sum_{t\in T}\sum_{n\in N}\sum_{i\in I_{n}}\sum_{u\in U}r_{n_{i},u}\left(t\right)\\ =\sum_{t\in T}\sum_{n\in N}\sum_{i\in I_{n}}\sum_{u\in U}x_{n_{i},u}(t)y_{u,n_{i}}(t)W_{u}\log\left(1+\frac{p_{u,n_{i}}(t)g_{n_{i},u}(t)}{\sum_{u^{\prime }\in U\setminus \{u\}}p_{u^{\prime },n_{i}}(t)g_{n_{i},u^{\prime }}(t)+\sigma^{2}}\right)\\ =\sum_{t\in T}\sum_{n\in N}\sum_{i\in I_{n}}\sum_{u\in U}x_{n_{i},u}(t)y_{u,n_{i}}(t)W_{u}\log\left(1+\frac{p_{u,n_{i}}(t)h_{n_{i},u}(t)}{\sum_{u^{\prime }\in U\setminus \{u\}}p_{u^{\prime },n_{i}}(t)h_{n_{i},u^{\prime }}(t)+\sigma^{2}d_{n_{i},u}^{\alpha }(t)}\right) \end{array}$$

(35a) s.t. C1–C7,

(35b) $$C16:\log\left(1+\frac{B_{u,n_{i}}\left(t\right)}{\Gamma_{n_{i},u}\left(t\right)}\right)⩾R^{th}$$

where $B_{u,n_{i}}\left(t\right)=p_{u,n_{i}}\left(t\right)h_{n_{i},u}\left(t\right)$, and $\Gamma_{n_{i},u}\left(t\right)=\Gamma_{n_{i},u}\left(t\right)=\sum_{u^{\prime }\in U\backslash \{u\}}p_{u^{\prime },n_{i}}\left(t\right)h_{n_{i},u^{\prime }}\left(t\right)+\sigma^{2}d_{n_{i},u}{\left(t\right)}^{\alpha }$. Note that, due to the non-convexity of constraints C5 and C7, the UAV trajectory optimization problem is neither a concave optimization problem nor a quasi-concave maximization problem. As a result, it is generally difficult to obtain the global optimum using existing methods, and no efficient solution method currently exists. To address this issue, we adopt the SCA technique. Specifically, we first introduce an auxiliary variable set $H=\left\{h_{n_{i}}(t),i\in I\right\}$ to approximate and reformulate the original problem for iterative solution, i.e.,

(36) $$C17:h_{n_{i}}\left(t\right)⩽\frac{1}{{\left({\left\|l_{u}^{\prime }(t)-l_{n_{i}}^{\prime }(t)\right\|}^{2}+H^{2}\right)}^{\alpha /2}},\;\forall i\in I$$

Therefore, the original UAV trajectory optimization problem, denoted as sub−TO, can be reformulated as the following problem.

(37) $$\begin{array}{l} sub-TO_{1}:\underset{l,h}{\max}\sum_{t\in T}\sum_{n\in N}\sum_{i\in I_{n}}\sum_{u\in U}r_{n_{i},u}(t)\\ =\sum_{t\in T}\sum_{n\in N}\sum_{i\in I_{n}}\sum_{u\in U}x_{n_{i},u}(t)y_{u,n_{i}}(t)W_{u}\log\left(1+B_{u,n_{i}}(t)h_{u,n_{i}}(t)\right) \end{array}$$

Since the above optimization problem is also non-convex, we employ the SCA method to obtain a linear lower-bound approximation. Let $f\left(l_{u}^{\prime }(t)\right)=\frac{1}{{\left({\left\|l_{u}^{\prime }(t)-l_{n_{i}}^{\prime }(t)\right\|}^{2}+H^{2}\right)}^{\alpha /2}}$, the function is neither globally convex nor concave; however, it can be approximated near a given point $l_{u}^{\prime (\varpi )}\left(t\right)$ using a first-order Taylor expansion, as shown below.

(38) $$f\left(l_{u}^{\prime }(t)\right)⩾f\left(l_{u}^{\prime (\varpi )}(t)\right)+\nabla f{\left(l_{u}^{\prime (\varpi )}(t)\right)}^{T}\left(l_{u}^{\prime }(t)-l_{u}^{\prime (\varpi )}(t)\right)$$

where $f\left(l_{u}^{\prime (\varpi )}(t)\right)=\frac{1}{{\left({\left\|l_{u}^{\prime (\varpi )}(t)-l_{n_{i}}^{\prime }(t)\right\|}^{2}+H^{2}\right)}^{\alpha /2}}$,$\nabla f=-\alpha \frac{l_{u}^{\prime }\left(t\right)-l_{n_{i}}^{\prime }\left(t\right)}{{\left({\left\|l_{u}^{\prime (\varpi )}\left(t\right)-l_{n_{i}}^{\prime }\left(t\right)\right\|}^{2}+H^{2}\right)}^{\frac{\alpha }{2}+1}}$.Therefore, the original constraint can be approximated by its lower bound, i.e.,

(39) $$C18:h_{n_{i}}\left(t\right)⩽f\left(l_{u}^{\prime (\varpi )}(t)\right)+\nabla f{\left(l_{u}^{\prime (\varpi )}(t)\right)}^{T}\left(l_{u}^{\prime }(t)-l_{u}^{\prime (\varpi )}(t)\right),\;\forall i\in I$$

Similarly, constraint C5 is non-convex. To address this, we define $L^{\varpi }\;=\;\left\{l_{u}^{\varpi }\left(t\right)\right\}$ as the trajectory obtained at the ϖth iteration. Then, by applying the first-order Taylor expansion, constraint C5 can be relaxed and approximated as follows.

(40) $${\left\|l_{u}^{\prime }\left(t\right)-p_{k}\right\|}^{2}⩾2{\left(l_{u}^{\prime (\varpi )}\left(t\right)-p_{k}\right)}^{T}\times \left(l_{u}^{\prime (\varpi )}\left(t\right)-l_{u}^{\prime }\left(t\right)\right)+{\left\|p_{k}-l_{u}^{\prime (\varpi )}\left(t\right)\right\|}^{2}\overset{\Delta }{=}C_{u,k}^{\varpi }\left(t\right)$$

Therefore, constraint C5 is transformed into constraint $C19:C_{u,k}^{\varpi }\left(t\right)⩾r_{NFZ}^{2}$, which is convex. The equation $C_{u,k}^{\varpi }\left(t\right)⩾r_{NFZ}^{2}$ defines a line (a hyperplane in 2D) that is tangent to the NFZ circle at the point where the line connecting $p_{k}$ and $l_{u}^{\prime (\varpi )}(t)$ intersects the circle. The inequality $C_{u,k}^{\varpi }\left(t\right)⩾r_{NFZ}^{2}$ then specifies the half-space that *excludes* the NFZ, effectively replacing the circular keep-out zone with a linear keep-out boundary at each iteration. Since the Taylor expansion provides a lower bound for the convex quadratic function ${\left\|l_{u}^{\prime }\left(t\right)-p_{k}\right\|}^{2}$, this approximation ensures that the solution at iteration ϖ+1 will strictly satisfy the original NFZ constraint if the reference point $l_{u}^{\prime \left(\varpi \right)}\left(t\right)$ itself is feasible. The UAV trajectory optimization subproblem $sub-TO_{1}$ can be transformed into problem $sub-TO_{2}$.

(41) $$\begin{array}{l} sub-TO_{2}:\underset{l,h}{\max}\sum_{t\in T}\sum_{n\in N}\sum_{i\in I_{n}}\sum_{u\in U}r_{n_{i},u}(t)\\ =\sum_{t\in T}\sum_{n\in N}\sum_{i\in I_{n}}\sum_{u\in U}x_{n_{i},u}(t)y_{u,n_{i}}(t)W_{u}\log\left(1+B_{u,n_{i}}(t)\cdot h_{u,n_{i}}(t)\right) \end{array}$$

(41a) s.t.    C1–C4, C6–C7, C19

Through the above transformations and approximations, the UAV trajectory optimization problem $sub-TO_{2}$ is converted into a convex optimization problem that can be efficiently solved using standard convex optimization solvers such as CVX [40]. In Algorithm 2, we present the pseudocode for UAV trajectory optimization based on SCA and analyze its computational complexity.

**Algorithm 2:** UAV Trajectory Optimization Algorithm Based on SCA.1:Input: User Association Strategy $x_{i,j}\left(t\right)$, Slice Resource Allocation Ratio $b_{n}(t)$,$f_{n}(t)$, Initial trajectory $L^{\left(0\right)}=\left\{l_{u}^{\prime (0)}(t)\right\}$, NFZ’s Information $p_{k}$ and $r_{NFZ}$, Maximum Iterations $\varpi_{\max}$;2:Output: Optimized UAV trajectory $L^{*}=\left\{l_{u}^{\prime *}(t)\right\}$3:  Initialize iteration index ϖ←0;4:  Repeat:5:   **For** the ϖ-th iteration, given reference trajectory $L^{\left(\varpi \right)}$
6:      Solve the convex optimization problem (sub-TO_2_):7:     Let $L^{\left(\varpi +1\right)}=\left\{l_{u}^{\prime \left(\varpi +1\right)}(t)\right\}$ be the optimal solution of the above problem;8:     Update ϖ←ϖ+1;9:     Until $\varpi >\varpi_{\max}$
10:     Return $L^{*}=L^{\left(\varpi \right)}$


In this study, we optimize the UAV trajectory using SCA and CVX. According to [24], the computational complexity of CVX is $O\left({\left(U\varpi \right)}^{3.5}\right)$.

### 5.3. Resource Allocation (RA)

We propose a reinforcement learning algorithm based on Soft Actor–Critic (SAC) to solve the resource allocation problem. SAC is a deep reinforcement learning algorithm that is well-suited for continuous action spaces. It is built upon the maximum entropy reinforcement learning framework and aims to maximize the expected cumulative reward by introducing an entropy term as a regularizer. This encourages exploration in the policy space to find the optimal policy μ. In the SAC algorithm, the Markov Decision Process (MDP) is defined by the tuple $\left(S,A,R\right)$ [41], where the state space S and the action space A are continuous, and R denotes the reward function. The three key components of the MDP are defined as follows.

(1) State Space: In this system, the allocation decisions for bandwidth and computing resources are influenced by the number and size of data packets within each slice. Therefore, the system state at each time slot is defined as the number of data packets that each slice needs to transmit, i.e., $s(t)=\left\{packet_{n}\left(t\right),n\in N\right\}$.

(2) Action Space: The action space includes two optimization variables, defined as $a(t)=\left\{b_{n}\left(t\right),f_{n}\left(t\right),n\in N\right\}$. At time slot t, the agent selects an action a(t) from the action space to make a decision.

(3) Reward: The reward is a function of the state and action, reflecting the quality of an action taken in a given state. To effectively solve the optimization problem, we define the reward function as follows

(42) $$\begin{array}{l} r(t)=\xi_{1}\left(U_{\mathrm{renu}}(t)-U_{\cos t}(t)\right)-\xi_{2}\sum_{i\in I}\max\left\{R_{e}-r_{n_{i},j}(t),0\right\}\\ \;\;\;\;\;\;\;-\xi_{3}\max\left\{T_{n_{i},j}(t)-\varphi_{n}^{\mathrm{comp}}(t),0\right\} \end{array}$$

The purpose of designing this reward function is to maximize system utility while ensuring a minimum rate threshold and reducing computational bottlenecks. The function consists of three components: the first term represents the difference between system revenue and cost; the second term is a penalty for insufficient data rate, ensuring that each user’s rate requirements are met; and the third term reflects the deviation between actual latency and the slice’s tolerable delay, serving as a penalty for delay violations. These three components are balanced using weighting factors $\xi_{1},\xi_{2}$, and $\xi_{3}$ to comprehensively account for different optimization objectives.

The goal of SAC is not solely to maximize the expected return, but to maximize the expected return while also maximizing the entropy of the policy. To address the problem of resource allocation in network slicing, the resource allocation algorithm based on Soft Actor–Critic (SAC-RA) defines a maximum entropy objective formulated as follows.

(43) $$J\left(\mu \right)=\sum_{t=1}^{T}E_{\left(s(t),a(t)\right)∼\rho_{\mu }}\left[r\left(s(t),a(t)\right)-\tilde{\alpha }\log\left(\mu (\cdot |s(t))\right)\right]$$

where $\tilde{\alpha }$ is the temperature parameter that balances the trade-off between reward maximization and entropy maximization, and $\rho_{\mu }$ represents the state-action distribution under the current policy μ. In SAC-RA, the parameters $\beta_{1}$ and $\beta_{2}$ of the Q-function are updated at fixed time intervals by minimizing the soft Bellman residual [42].

(44) $$J_{Q}\left(\beta_{i}\right)=E_{\left(s(t),a(t)\right)\sim D}\left[\frac{1}{2}{\left(Q_{\beta_{i}}\left(s(t),a(t)\right)-\hat{y}(t)\right)}^{2}\right]$$

where $\hat{y}(t)=\left(r\left(t\right)+\gamma \left(\underset{\kappa =1,2}{\min}Q_{{\bar{\beta }}_{\kappa }}\left(s\left(t+1\right),a\left(t+1\right)\right)-\tilde{\alpha }\log\mu_{\varphi }\left(a\left(t+1\right)|s\left(t+1\right)\right)\right)\right)$. The parameter of policy function φ is updated by

(45) $$J_{\mu }\left(\varphi \right)=E_{s(t)∼D,\varepsilon (t)\in T}\left[\begin{array}{l} \tilde{\alpha }\log\mu_{\varphi }\left(f_{\varphi }\left(\varepsilon (t);s(t)\right)|s(t)\right)\\ -\underset{\kappa =1,2}{\min}Q_{\beta_{\kappa }}\left(s(t),f_{\varphi }\left(\varepsilon (t);s(t)\right)\right) \end{array}\right]$$

**Figure 5:**
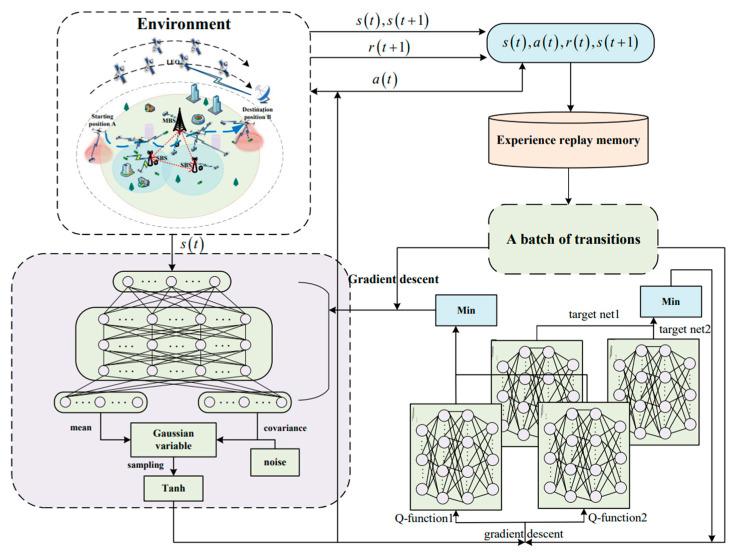
Sketch of SAC-RA.

In the above expression, $f_{\varphi }\left(\varepsilon (t);s(t)\right)$ is a reparameterization function [43], which maps the state s(t) and noise ε(t) to the action a(t). Specifically, given a state s(t), the action is computed through $a\left(t\right)=f_{\varphi }\left(\varepsilon (t);s(t)\right)$. The overall framework of SAC-RA is illustrated in Figure 5, and the resource allocation algorithm SAC-RA is shown in Algorithm 3.

**Algorithm 3:** SAC-RA Algorithm.1:Initialize: policy network φ, Q-functions $\beta_{1}$ and $\beta_{2}$, Parameters of target networks ${\bar{\beta }}_{1}\leftarrow \beta_{1},{\bar{\beta }}_{2}\leftarrow \beta_{2}$, replay buffer D, learning rate λ, discount factor γ, entropy coefficient $\tilde{\alpha }$.2: **for** each episode **do**3: Initialize state s(t);4: **for** step t = 1 to T**do**5:    Selects action $a\left(t\right)$ according to $\mu \left(a\left(t\right)|s\left(t\right)\right)$;6:    Execute action a(t), and observe the immediate reward r(t) and the     state $s\left(t+1\right)$
7:   Store $\left(s\left(t\right),a\left(t\right),r\left(t\right),s\left(t+1\right)\right)$ in the experience replay buffer D;8:   **if** replay buffer size ≥ batch size **then**9:     Randomly sample batch $\left[s\left(t\right),a\left(t\right),r\left(t\right),s\left(t+1\right)\right]$ from buffer D;10:     Calculate the target Q-value, i.e., Equation (43);11:     Update Q Network: $\beta_{i}\leftarrow \beta_{i}-\lambda \nabla_{\beta_{i}}J_{Q}\left(\beta_{i}\right);$
12:     Update policy-function parameter: i.e., Equation (44), $\varphi \leftarrow \varphi -\lambda \nabla_{\varphi }J_{\mu }\left(\varphi \right)$;13:    Update the target network using soft updates: ${\bar{\beta }}_{i}\leftarrow \tau \beta_{i}+\left(1-\tau \right){\bar{\beta }}_{i}$;14:   **end if**15:  **end for**16:**end for**

We assume that the Actor and Critic networks in SAC are Z-layer fully connected neural networks, with $Y_{Z}$ denoting the number of neurons in the Z-th layer. Thus, the forward/backward propagation cost of the neural networks is $\sum_{z=0}^{Z-1}Y_{Z}\times Y_{Z+1}$. Let $\left|N_{b}\right|$ be the batch size and $N_{Episode}$ the total number of training epochs. Accordingly, the computational complexity of the SAC-based slice resource allocation algorithm is $O\left(\left(\sum_{z=0}^{Z-1}Y_{Z}\times Y_{Z+1}\right)\times \left|N_{b}\right|\times N_{Episode}\right)$. Based on the computational complexities of each algorithm discussed above, the overall computational complexity of the proposed SAC-MS algorithm is $O\left(K\left[O\left(JI\log\left(JI\right)\right)+O\left({\left(U\varpi \right)}^{3.5}\right)+O\left(\left(\sum_{z=0}^{Z-1}Y_{Z}\times Y_{Z+1}\right)\times \left|N_{b}\right|\times N_{Episode}\right)\right]\right)$.

## 6. Simulation Results and Analysis

In this section, we first configure the key parameters for the experiments. Then, through a series of simulation experiments, we systematically evaluate the proposed algorithms, including hyperparameter analysis, environment parameter analysis, and comparisons with benchmark algorithms.

### 6.1. Simulation Setup

We verify the performance of the proposed SAC-MS algorithm through simulations implemented using Python 3.11.9 and PyTorch 2.3.0. The network topology is configured over a 600 m × 600 m area, including one macro base station (MBS), three small base stations (SBSs), 100 ground users, and two no-fly zones. The shadow fading for the MBS and SBS is set to 8 dB and 10 dB, respectively [44]. The additive white Gaussian noise power $\sigma^{2}$ is set to −174 dBm, and the carrier frequency $f_{c}$ is 2 GHz. The UAV operates at a fixed altitude of 80 m, with a maximum speed of 15 m/s and a minimum speed of 6 m/s. For the reinforcement learning setup, the learning rates of the actor and critic networks are set to 0.001 and 0.0015, respectively. The batch size is 128, the temperature coefficient is 0.001, and the total number of training iterations is 6000. We provide the training parameters of the experiments and summarize them in Table 4.

### 6.2. Parameter Analysis

#### 6.2.1. Analysis of Hyperparameters

In reinforcement learning, the choice of hyperparameters significantly affects the convergence behavior, learning stability, and overall performance of the algorithm. To ensure the reliability and robustness of the proposed SAC-MS algorithm, we conduct a systematic analysis of key hyperparameters, including the learning rates of the actor and critic networks, the batch size, and the temperature coefficient.

**Figure 6:**
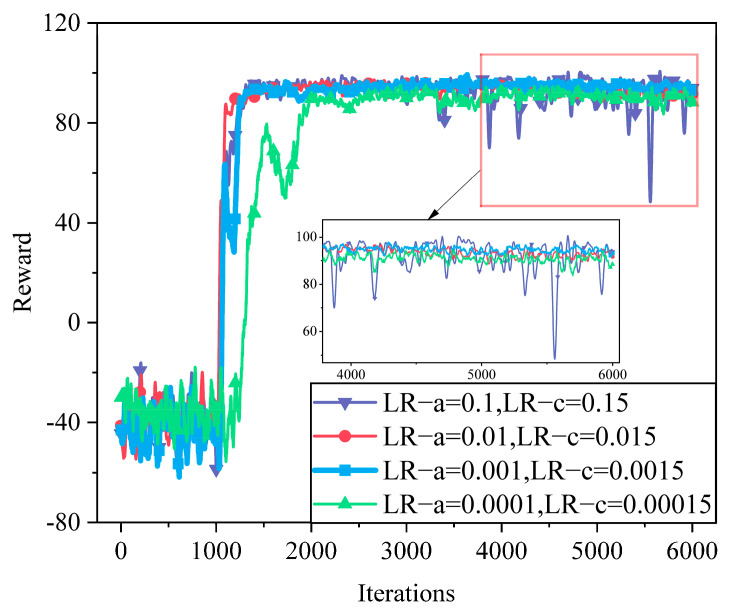
Convergence of different learning rates.

We conduct simulations using different learning rates. As shown in Figure 6, setting the actor and critic learning rates to 0.1 and 0.15 leads to strong reward oscillations, indicating unstable updates and poor convergence. When reduced to 0.01 and 0.015, the algorithm achieves faster initial learning but converges to a lower reward. In contrast, smaller rates (0.001 and 0.0015) result in slower initial progress but lead to more stable convergence and better final performance. This highlights the trade-off between convergence speed and stability in learning rate selection.

**Figure 7:**
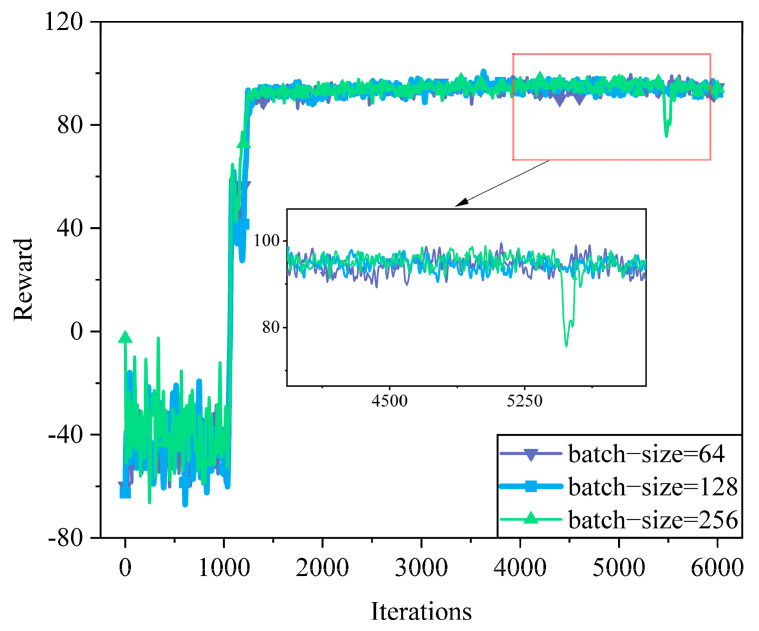
Convergence of different batch sizes.

Figure 7 compares the reward convergence under different batch sizes: 64, 128, and 256. All settings show rapid reward growth after initial exploration. The batch-size = 256 achieves faster early convergence but suffers from a noticeable performance drop around iteration 5400, indicating potential instability due to over-smoothed gradients. The batch-size = 64 yields more stable but slower learning due to high variance in updates. In contrast, batch-size = 128 provides the best trade-off, ensuring fast and stable convergence without significant fluctuations. This demonstrates that a moderate batch size (128) leads to more reliable training dynamics in SAC-MS.

**Figure 8:**
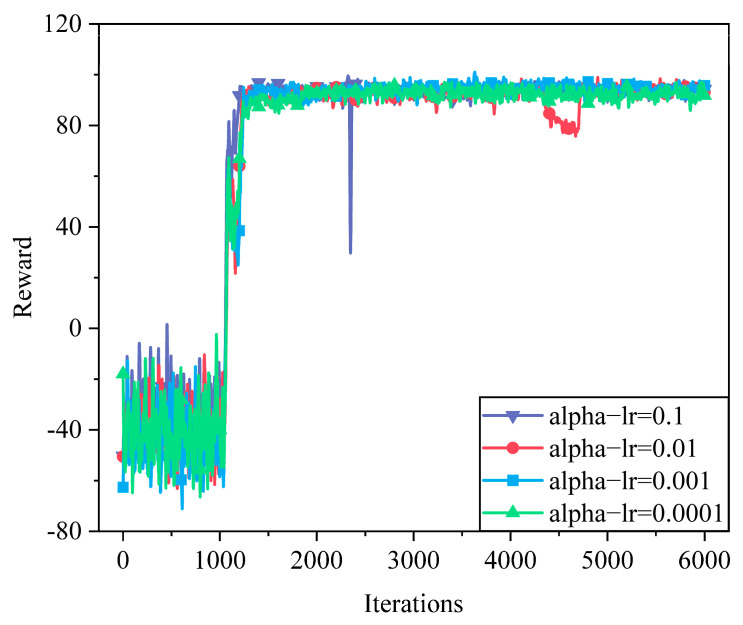
Convergence of different temperature coefficients.

As shown in Figure 8, different entropy temperature learning rates exhibit distinct convergence behaviors. A large alpha-lr (e.g., 0.1 and 0.01) results in rapid initial learning but introduces significant instability during training due to excessive entropy adjustments. Conversely, smaller values (e.g., 0.001 and 0.0001) provide smoother and more stable learning curves, ensuring consistent policy improvement. However, when the entropy temperature coefficient is too small (e.g., 0.0001), the convergence speed becomes relatively slow.

#### 6.2.2. Analysis of Environmental Parameters

**Figure 9:**
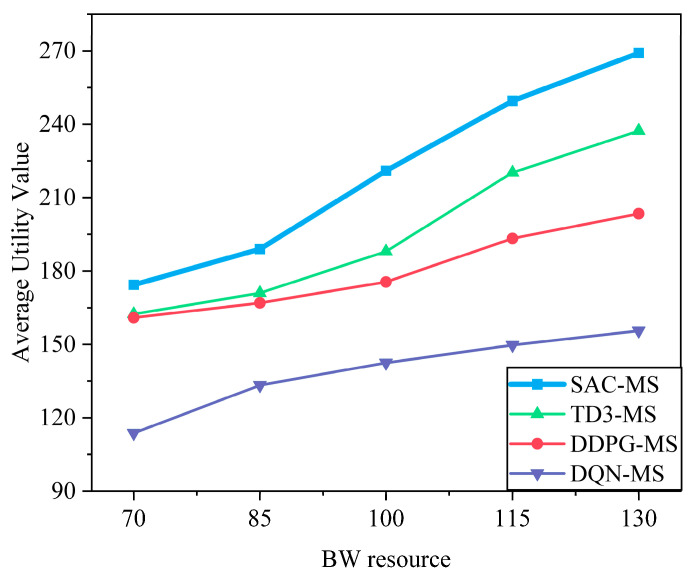
Impact of bandwidth on system utility. The utility of all algorithms increases with more bandwidth but exhibits diminishing returns. Once bandwidth becomes sufficient to meet user demands, performance saturates. The proposed SAC-MS algorithm consistently outperforms benchmarks across all bandwidth values.

**Figure 10:**
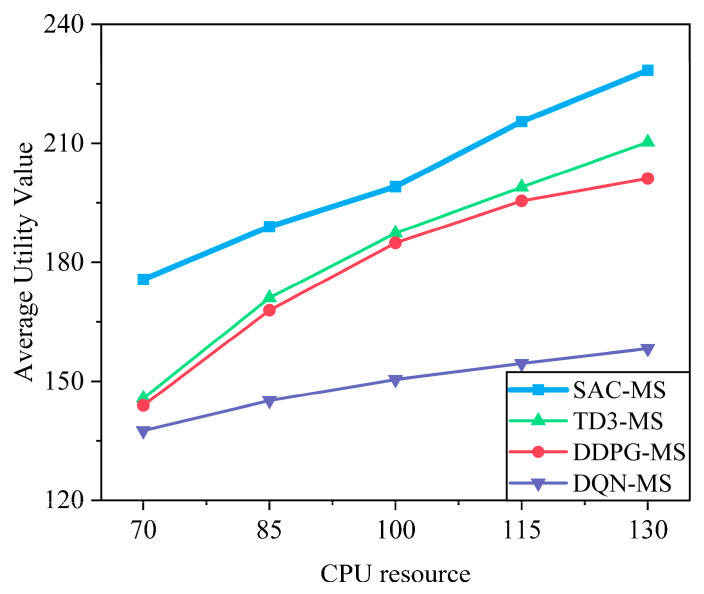
Impact of computing resource on system utility. Similarly to bandwidth, increased computing resources improve utility, but the gains diminish after a point due to resource saturation. SAC-MS achieves the highest utility by more efficiently allocating these resources to meet heterogeneous task demands.

From Figure 9 and Figure 10, it can be observed that as bandwidth and computational resources increase, the system utility generally rises monotonically and gradually exhibits diminishing marginal returns. When resources are scarce (e.g., in the 70–100 range), system utility grows rapidly; as resources become sufficient (e.g., in the 115–130 range), the growth rate decreases, and system performance tends to saturate. It should be noted that in certain intervals (e.g., 100–115), the increase appears relatively larger. This is because the simulation results are based on discrete sampling points, and the additional resources in this interval happen to satisfy the service requirements of some users, resulting in a temporary improvement. Such local fluctuations do not alter the overall trend: as resources increase, the growth of system utility gradually slows and approaches saturation.

**Figure 11:**
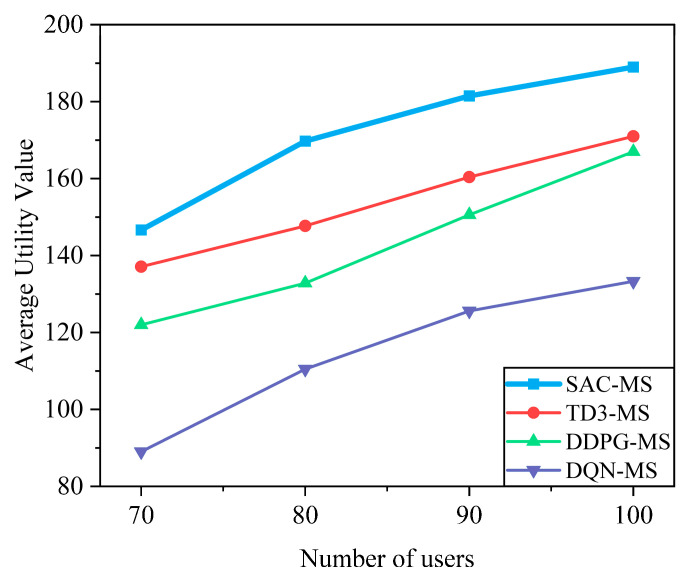
Impact of the number of users on the system utility. System utility grows with the number of users since more users lead to more efficient utilization of bandwidth and computing resources. However, beyond a certain point, resources become a bottleneck: interference increases and scheduling complexity rises, causing the growth rate of system utility to slow down. This illustrates the trade-off between multi-user diversity gain and resource limitation.

As shown in Figure 11, the system utility of all algorithms increases with the number of users. However, the superior efficiency of the proposed SAC-MS algorithm allows it to fully utilize the available resources, causing its growth to significantly slow down as it approaches the system capacity bottleneck at around 100 users. In contrast, the less efficient DDPG-MS and TD3-MS algorithms exhibit a more linear growth trend within the observed range, indicating that they have not yet fully stressed the system resources. This further demonstrates that SAC-MS can achieve higher resource utilization efficiency.

**Figure 12:**
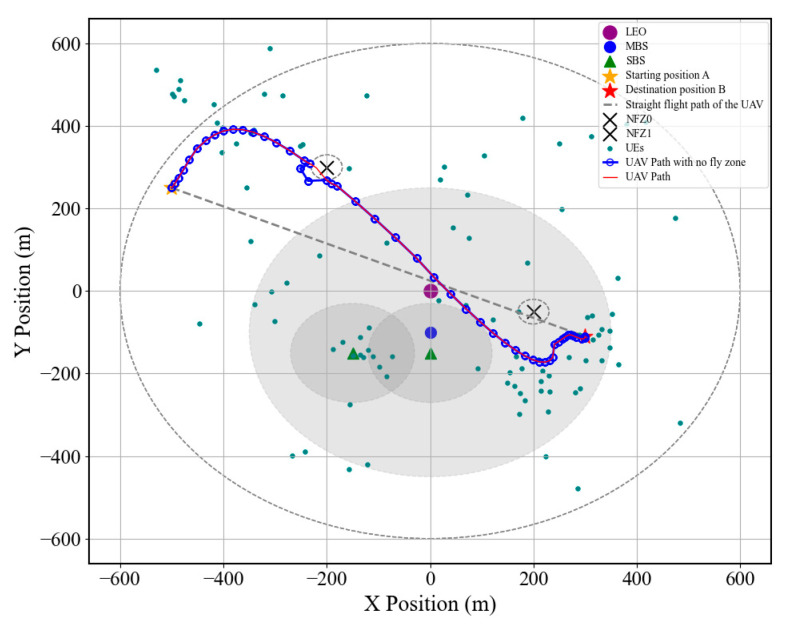
UAV trajectory diagram under the no-fly zone constraint. The optimized UAV trajectory avoids NFZs while flying close to dense user areas. This reduces communication distance, enhances SNR, and improves throughput while ensuring safety and regulatory compliance. It highlights how NFZ-aware planning balances safety with communication efficiency.

Figure 12 illustrates the optimal UAV trajectory planning results under no-fly zone constraints. As shown in the figure, the UAV not only successfully avoids the designated NFZs, ensuring flight safety and regulatory compliance, but also navigates as close as possible to user-dense areas. Such a trajectory design significantly shortens the communication distance between the UAV and ground users, thereby improving the signal-to-noise ratio (SNR), enhancing data transmission rates, and reducing communication latency. Moreover, flying near regions with high user demand facilitates more efficient task execution and real-time resource allocation.

#### 6.2.3. Performance Comparison

**Figure 13:**
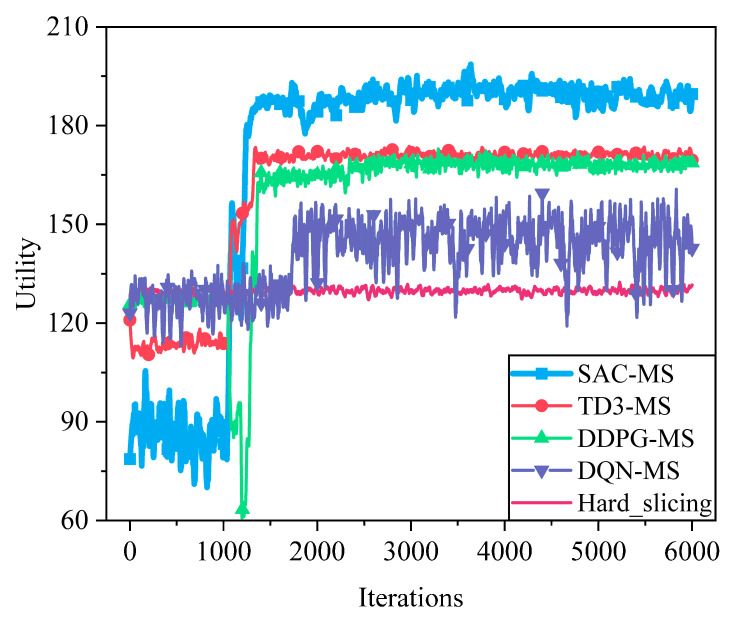
Impact of different algorithms on system utility. The proposed SAC-MS algorithm converges faster and to a higher average utility than the TD3-MS, DDPG-MS, and DQN-MS benchmarks. SAC’s superior performance is attributed to its maximum entropy framework, which encourages more explorative and stable policy learning in high-dimensional continuous action spaces, avoiding the overestimation bias and training instability common in DDPG and TD3.

Figure 13 illustrates the comparison of system utility achieved by different algorithms in the resource allocation task. As shown in the figure, the SAC algorithm converges rapidly after approximately 1000 training iterations and consistently maintains the highest utility throughout the entire training process, demonstrating its superior policy representation capability and efficient exploration performance in continuous action spaces. TD3 and DDPG follow in terms of performance, with TD3 showing better convergence stability than DDPG due to its delayed update mechanism and double Q-network structure. In contrast, DQN adopts a discrete action space, resulting in coarse-grained resource allocation and poor adaptability to dynamic environments, leading to significant fluctuations in system utility. Moreover, the hard slicing strategy does not incorporate any learning mechanism and thus cannot dynamically optimize according to environmental changes, resulting in the lowest utility level. These results further highlight the advantages of continuous action space-based deep reinforcement learning methods SAC in complex and high-dimensional resource allocation tasks.

**Figure 14:**
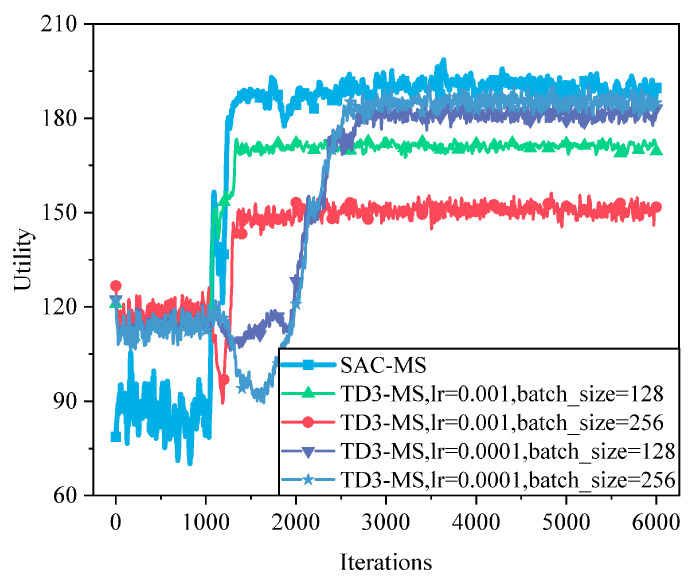
Convergence performance of the TD3 algorithm based on different hyperparameters. Although careful tuning (e.g., lr = 0.0001, batch_size = 256) stabilizes TD3, SAC still outperforms TD3 by ~2.2%. The advantage arises from SAC’s entropy-regularized objective, which avoids premature convergence to suboptimal policies and encourages diverse exploration.

As shown in Figure 14, the performance of TD3 under different hyperparameter settings is illustrated. Compared to TD3, SAC employs a maximum entropy framework, which enhances exploration efficiency and helps avoid local optima. In terms of system utility, SAC achieves approximately a 2.2% improvement over TD3’s best hyperparameter configuration (lr = 0.0001, batch_size = 256).

**Figure 15:**
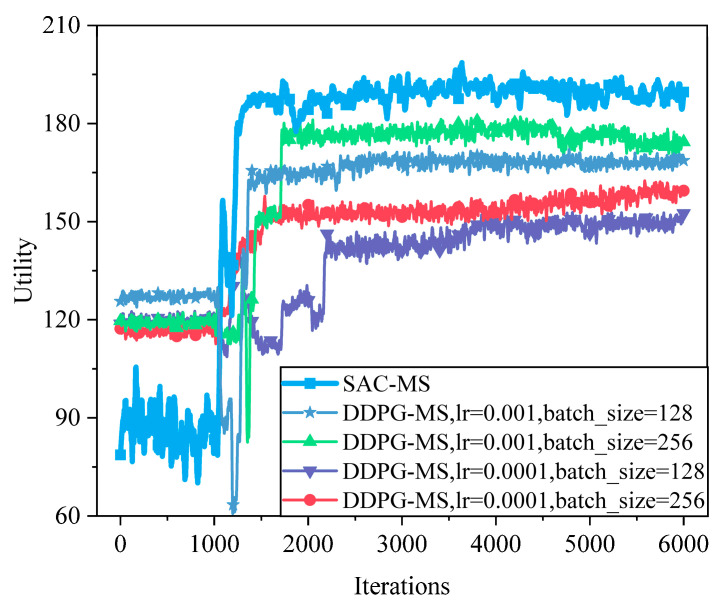
Convergence performance of the DDPG algorithm based on different hyperparameters. Even under its best hyperparameter settings (lr = 0.001, batch_size = 256), DDPG lags behind SAC. SAC converges faster and improves utility by ~7.8%, largely because it mitigates Q-value overestimation and handles high-dimensional continuous tasks more robustly.

As shown in Figure 15, the performance of the DDPG-based algorithm under different hyperparameters is illustrated. Compared to DDPG, SAC demonstrates faster convergence, addresses the Q-value overestimation problem in DDPG, and is better suited for high-dimensional tasks. Relative to DDPG’s best hyperparameter combination (lr = 0.001, batch_size = 256), SAC achieves a 7.8% improvement in system utility.

**Figure 16:**
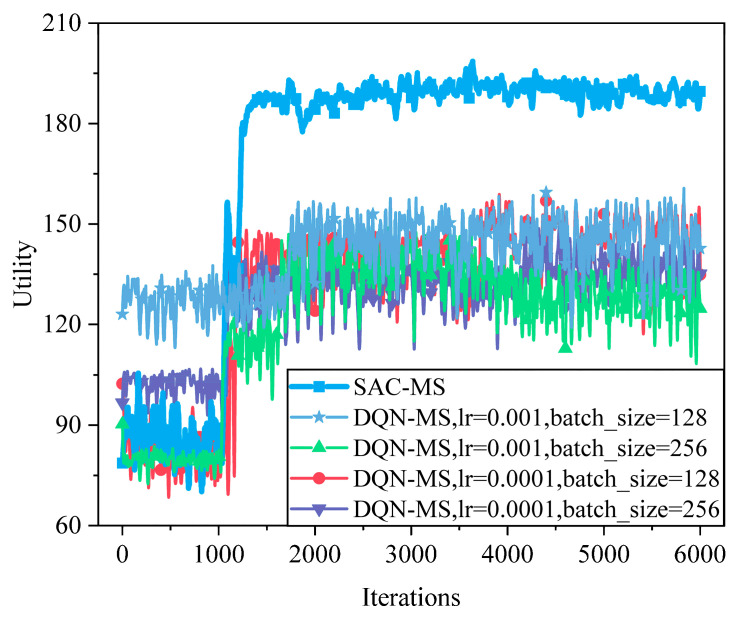
Convergence performance of the DQN algorithm based on different hyperparameters. DQN shows unstable convergence and poor adaptability because its discrete action space limits fine-grained resource allocation. Compared with DQN’s best case, SAC improves system utility by ~31.25%, highlighting the importance of continuous action learning.

Figure 16 compares DQN variants under different hyperparameter settings. DQN is suitable for discrete action spaces; however, when applied to continuous control tasks, it typically suffers from slow convergence, large fluctuations, and difficulty in achieving optimal performance. As illustrated in the figure, SAC outperforms DQN’s best hyperparameter configuration (lr = 0.001, batch_size = 128) by achieving a 31.25% improvement in system utility.

**Figure 17:**
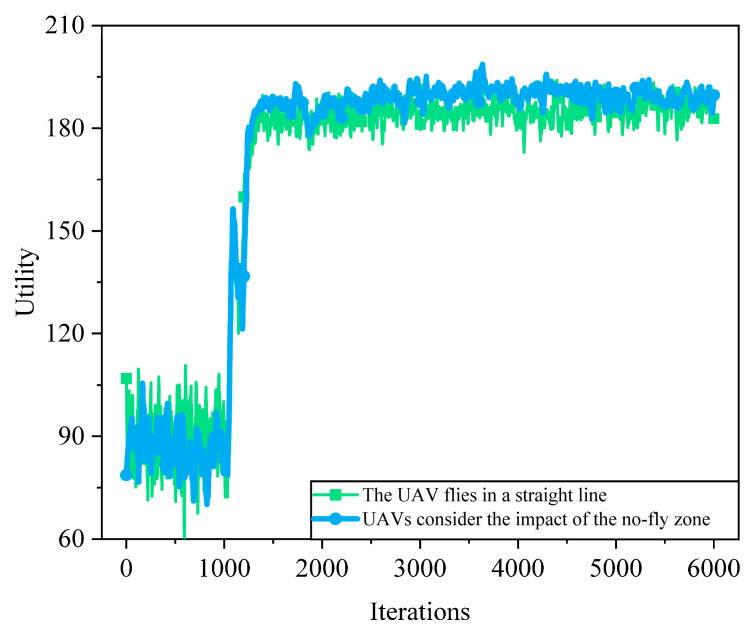
System utility comparison with and without NFZs. SAC-MS generates smoother and shorter UAV trajectories that simultaneously avoid NFZs and minimize communication distance. Baseline methods either detour excessively or approach NFZ boundaries too closely, demonstrating SAC-MS’s ability to balance safety and efficiency.

Figure 17 illustrates the trend of system utility over training iterations under different UAV flight strategies. It can be observed that when the UAV flight strategy accounts for no-fly zone constraints, the system utility is significantly higher compared to the case without such consideration. This is because no-fly zones act as hard constraints that limit the feasible flight region of the UAV. If these zones are not effectively avoided during path planning, the UAV may traverse illegal areas, resulting in communication interruptions, invalid trajectories, or resource waste. In contrast, flight strategies that consider no-fly zones can proactively avoid restricted areas and plan feasible paths, thereby ensuring communication link continuity and service quality, ultimately improving resource allocation efficiency and overall system performance.

**Figure 18:**
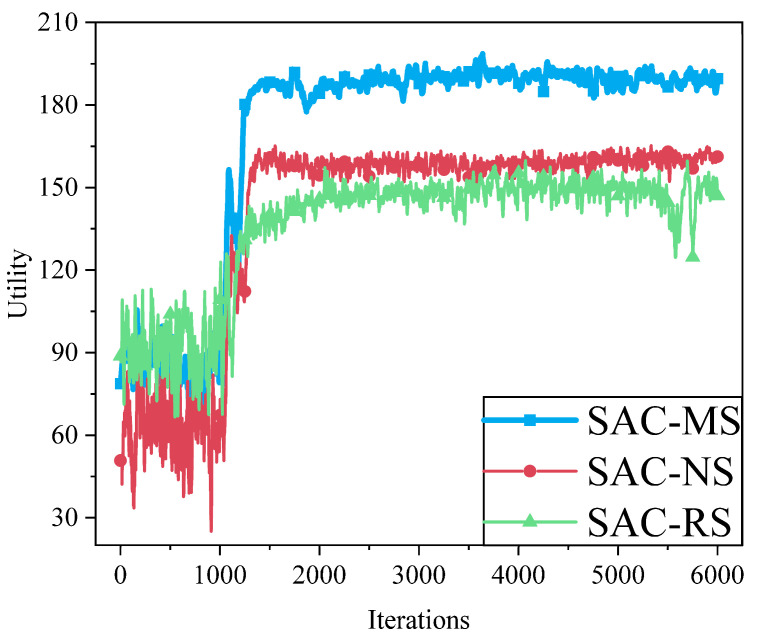
Comparison of different user association methods. The proposed matching game-based method (SAC-MS) significantly outperforms both nearest-station (SAC-NS) and random association (SAC-RS) strategies. This demonstrates that an intelligent association strategy, which dynamically balances user-channel quality and base-station load, is crucial for maximizing overall system performance, rather than simply connecting to the nearest node or making random choices.

To evaluate the impact of different user association strategies on system performance. Figure 18 compares three schemes: matching game-based association (SAC-MS), nearest-distance association (SAC-NS), and random association (SAC-RS). The figure illustrates the trend of utility values over training iterations. The results show that SAC-MS consistently achieves the highest utility, eventually stabilizing around 189, demonstrating superior system performance. This is mainly attributed to the matching game’s two-sided preference model, which dynamically matches users with base stations by considering both channel conditions and base station capacity, effectively avoiding base station overload and resource wastage, thereby enhancing overall system utility. In contrast, SAC-NS converges quickly in the early stages but considers only distance while ignoring interference and load. SAC-RS, which randomly selects base stations without considering channel state or user demand, shows poor convergence and large fluctuations, with a final utility of only around 150-indicating the weakest performance.

## 7. Discussion and Future Direction

The proposed SAC-MS framework provides a theoretical foundation for joint resource optimization in space–air–ground integrated networks (SAGINs) and demonstrates significant performance improvements in simulations. However, several challenges and limitations remain when moving from theoretical modeling to practical deployment, which also point to promising future research directions.

First, the limited battery capacity of UAVs constrains flight endurance and service coverage, suggesting that incorporating an energy consumption model into trajectory optimization is a valuable extension. Second, communication delays and signaling overhead in real SAGINs may weaken the timeliness of resource allocation, highlighting the need for delay-tolerant mechanisms and lightweight coordination protocols. Third, this study primarily focuses on a single-UAV scenario; although the proposed method can be extended to multi-UAV networks, the computational complexity of SCA-based trajectory optimization will significantly increase with the number of UAVs. Future work may explore distributed architectures and multi-agent reinforcement learning to enhance scalability and practicality. Fourth, the communication model relies on the assumption of perfect and instantaneous CSI, which is highly challenging to obtain in real environments with high mobility and severe fading. Developing robust optimization frameworks that can withstand CSI uncertainty and estimation errors will be an important direction. Fifth, although the proposed method has the potential to be extended to account for satellite orbital dynamics, in this study the satellite position is treated as fixed or only slowly varying over time. This limitation also provides new insights and directions for future research. Finally, the NFZs in this study are idealized as static cylindrical regions, whereas in reality they may be dynamic, irregularly shaped, or partially unknown, and may also involve multi-layer altitude restrictions. Future research could integrate real-time data from UAV traffic management (UTM) systems to enable trajectory planning under more complex and dynamic NFZ conditions.

## 8. Conclusions

To address challenges such as resource limitations caused by the exponential growth in user service demands, coverage blind spots in remote areas, and restrictions imposed by no-fly zones (NFZs), this paper investigated the joint optimization problem of user association, UAV trajectory planning, and network slicing resource allocation in space–air–ground integrated networks (SAGINs) under NFZ constraints. To facilitate the solution process, the original problem was decomposed into three subproblems: user association was solved using a many-to-one matching game; UAV trajectory was optimized via the sequential convex approximation (SCA) algorithm; and dynamic slice resource allocation was achieved through a deep reinforcement learning (DRL) framework (SAC). Simulation results demonstrated that, compared with benchmark algorithms, the proposed SAC-MS method more effectively satisfied users’ quality of service (QoS) requirements, avoided various no-fly zones, and achieved better resource allocation balance across network slices, thereby enhancing the overall system utility.

## Figures and Tables

**Table 1:** Summary of main notations.

Notation	Definition
L	The set of LEO satellite
B	The set of terrestrial base stations
U	The set of unmanned aerial vehicle
$I_{n}$	The set of users associated with slice
J	The set of all base stations
N	The set of network slice
K	The set of No-Fly Zone
W	The set of bandwidth resources
F	The set of computing resources
$x_{n_{i},j}\left(t\right)$	Association variable between user i and base station j on slice n
$b_{n,j}\left(t\right)$	Bandwidth allocation ratio for slice n
$f_{n,j}\left(t\right)$	Computation resource allocation ratio for slice n
$y_{j,n_{i}}\left(t\right)$	Bandwidth allocation ratio from base station j to user i on slice n
$\xi_{1},\xi_{2},\xi_{3}$	Weight coefficients
$r_{NFZ}$	No-fly zone radius
α	Path loss exponent
$\tilde{\alpha }$	Temperature coefficient
$\varphi_{n}^{comp}$	Latency tolerance threshold of slice n
$R_{e}$	Minimum transmission rate threshold

**Table 2:** Summary of Acronyms.

Acronyms	Definition
SAGIN	Space–Air–Ground Integrated Network
UAV	Unmanned Aerial Vehicle
LEO	Low Earth Orbit
TBS	Terrestrial Base Station
MBS	Macro Base Station
SBS	Small Base Station
NFZ	No-Fly Zone
SLS	Latency-Sensitive Slice
SLT	Latency-Tolerant Slice
SR	High-Data-Rate Slice
UA	User Association
TO	Trajectory Optimization
RA	Resource Allocation
SAC	Soft Actor–Critic
TD3	No-fly zone radius
DDPG	Deep Deterministic Policy Gradient
DQN	Deep Q-Network
SAC-MS	Matching game, Sequential Convex Approximation, and Soft Actor–Critic-based Multi-Slice optimization algorithm

**Table 3:** Literature comparison.

Related Paper	Objective	UAV	Network Slicing	NFZ	UAV Trajectory Optimization	User Association	Resource Allocation
[18]	minimize the maximum computation delay	√	×	×	√	×	√
[19]	Minimize the total system cost	√	×	×	×	×	√
[21]	Maximize the long term network utility	×	×	×	×	×	√
[23]	Maximize the minimum throughput	√	×	×	√	√	×
[24]	Minimize the system latency	√	×	×	√	×	√
[25]	Maximize the system EE	√	×	×	√	×	√
[26]	Minimize the system’s weighted energy consumption	√	×	×	×	×	√
[27]	SINR, average rate, and mobility-induced time overhead	×	×	×	×	√	×
[28]	Maximizing QoE of users	√	×	×	√	√	√
[29]	Maximum long-term overall network utility	×	×	×	×	√	√
[30]	Maximize the network utility reflecting proportional fairness	×	×	×	×	√	√
[31]	Weighting of maximized throughput, SINR and minimized delay	√	√	×	×	√	√
[32]	Minimize the weighted energy consumption	√	×	×	√	√	√
[33]	Maximize the residual energy of the satellite	√	×	×	×	√	×
Our work	Maximum long-term overall system utility	√	√	√	√	√	√

**Table 4:** Simulation parameters for system.

Training Parameters	Value
Number of terrestrial base stations	3
Number of UAV	1
Number of ground users	100
UAV flight altitude (m)	80
Noise Power $\sigma^{2}$(dBm)	−174
Learning rate of actor network LR_a	0.001
Learning rate of critic network LR_c	0.0015
discount factor γ	0.99
entropy coefficient $\tilde{\alpha }$	0.001
Target network soft update coefficient τ	0.005
Replay Buffer Capacity D	100,000
batch size	128
Max episode	6000
Convergence threshold	0.001
Activation function	Tanh

## Data Availability

The data used to support the findings of this study are available from the corresponding author upon request.
